# Human Spinal Oligodendrogenic Neural Progenitor Cells Enhance Pathophysiological Outcomes and Functional Recovery in a Clinically Relevant Cervical Spinal Cord Injury Rat Model

**DOI:** 10.1093/stcltm/szad044

**Published:** 2023-08-24

**Authors:** Katarzyna Pieczonka, Hiroaki Nakashima, Narihito Nagoshi, Kazuya Yokota, James Hong, Anna Badner, Jonathon C T Chio, Shinsuke Shibata, Mohamad Khazaei, Michael G Fehlings

**Affiliations:** Division of Genetics and Development, Krembil Brain Institute, University Health Network, Toronto, Ontario, Canada; Institute of Medical Science, Faculty of Medicine, University of Toronto, Toronto, Ontario, Canada; Division of Genetics and Development, Krembil Brain Institute, University Health Network, Toronto, Ontario, Canada; Department of Orthopaedic Surgery, Nagoya University Graduate School of Medicine, Nagoya, Japan; Division of Genetics and Development, Krembil Brain Institute, University Health Network, Toronto, Ontario, Canada; Department of Orthopaedics, Keio University, Minatro City, Tokyo, Japan; Division of Genetics and Development, Krembil Brain Institute, University Health Network, Toronto, Ontario, Canada; Department of Orthopaedic Surgery, Graduate School of Medical Sciences, Kyushu University, Fukuoka, Japan; Division of Genetics and Development, Krembil Brain Institute, University Health Network, Toronto, Ontario, Canada; Institute of Medical Science, Faculty of Medicine, University of Toronto, Toronto, Ontario, Canada; Division of Genetics and Development, Krembil Brain Institute, University Health Network, Toronto, Ontario, Canada; Institute of Medical Science, Faculty of Medicine, University of Toronto, Toronto, Ontario, Canada; Division of Genetics and Development, Krembil Brain Institute, University Health Network, Toronto, Ontario, Canada; Institute of Medical Science, Faculty of Medicine, University of Toronto, Toronto, Ontario, Canada; Electron Microscope Laboratory, Keio University School of Medicine, Tokyo, Japan; Division of Genetics and Development, Krembil Brain Institute, University Health Network, Toronto, Ontario, Canada; Division of Genetics and Development, Krembil Brain Institute, University Health Network, Toronto, Ontario, Canada; Institute of Medical Science, Faculty of Medicine, University of Toronto, Toronto, Ontario, Canada; Division of Neurosurgery and Spinal Program, Department of Surgery, University of Toronto, Toronto, Ontario, Canada

**Keywords:** spinal cord injury, neural stem progenitor cells, oligodendrocytes, myelination

## Abstract

Traumatic spinal cord injury (SCI) results in the loss of neurons, oligodendrocytes, and astrocytes. Present interventions for SCI include decompressive surgery, anti-inflammatory therapies, and rehabilitation programs. Nonetheless, these approaches do not offer regenerative solutions to replace the lost cells, fiber tracts, and circuits. Neural stem/progenitor cell (NPC) transplantation is a promising strategy that aims to encourage regeneration. However, NPC differentiation remains inconsistent, thus, contributing to suboptimal functional recovery. As such, we have previously engineered oligodendrogenically biased NPCs (oNPCs) and demonstrated their efficacy in a thoracic model of SCI. Since the majority of patients with SCI experience cervical injuries, our objective in the current study was to generate human induced pluripotent stem cell-derived oNPCs (hiPSC-oNPCs) and to characterize these cells in vitro and in vivo, utilizing a clinically relevant rodent model of cervical SCI. Following transplantation, the oNPCs engrafted, migrated to the rostral and caudal regions of the lesion, and demonstrated preferential differentiation toward oligodendrocytes. Histopathological evaluations revealed that oNPC transplantation facilitated tissue preservation while diminishing astrogliosis. Moreover, oNPC transplantation fostered remyelination of the spared tissue. Functional analyses indicated improved forelimb grip strength, gait, and locomotor function in the oNPC-transplanted rats. Importantly, oNPC transplantation did not exacerbate neuropathic pain or induce tumor formation. In conclusion, these findings underscore the therapeutic potential of oNPCs in promoting functional recovery and histopathological improvements in cervical SCI. This evidence warrants further investigation to optimize and advance this promising cell-based therapeutic approach.

## Graphical Abstract

**Graphical Abstract:**
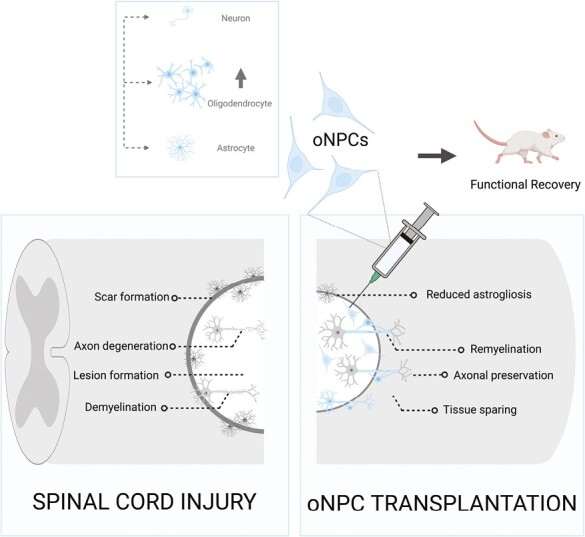

**Significance Statement**

Stem cell transplantation, of neural stem/progenitor cells (NPCs) in particular, is considered a promising treatment option for spinal cord injury (SCI), due to their ability to differentiate into oligodendrocytes, neurons, and astrocytes, which are known to be lost following injury. The replacement of lost oligodendrocytes and oligodendroglial precursor cells is important to promote functional recovery. However, the hostile microenvironment of the injured spinal cord often drives transplanted NPCs to give rise to a lower proportion of oligodendrocytes. To address this issue, we have developed and characterized oligodendrogenically biased NPCs (oNPCs) to promote oligodendrocyte differentiation while still allowing for trilineage differentiation into neurons and astrocytes. In this study, we transplanted oNPCs into a clinically relevant model of cervical contusion/compression SCI and confirmed their efficacy in improving pathophysiological and functional outcomes.

## Introduction

Traumatic spinal cord injury (SCI) is marked by an initial mechanical insult to the spinal cord. These mechanical forces cause oligodendrocyte necrosis, apoptosis, and necroptosis in the ensuing days, ultimately contributing to myelin degradation.^1,2^ Consequently, axons deficient in metabolic and protective support from the associated myelin sheath become susceptible to Wallerian degeneration, resulting in the deterioration of neurons that may have initially been spared during the primary traumatic mechanical insult.^3^ Moreover, oligodendrocytes also serve other supportive functions for neurons through the secretion of neurotrophic factors, immunomodulation, maintenance of the blood-brain barrier, and neurotransmitter regulation, thus making oligodendrocyte replacement an important therapeutic target for SCI.^4-7^

Despite considerable progress in the availability of pharmacological, surgical, and rehabilitative interventions, these approaches are incapable of sufficiently targeting the degenerative consequences of SCI. In this regard, cell-based strategies using neural stem/progenitor cells (NPCs) represent a relevant regenerative approach to restore lost or damaged neural cells. NPCs are particularly relevant for SCI because they are tripotent cells that differentiate into oligodendrocytes, in addition to neurons and astrocytes. However, after SCI, the microenvironment of the tissue prevents transplanted NPCs from differentiating into oligodendrocytes. While some methods, such as using oligodendrocyte precursor cells (OPCs) or glial restricted progenitors (GRPs), can increase the efficacy of oligodendrocyte differentiation, these methods do not replace the lost neurons that are necessary for relaying signals and achieving optimal functional recovery.^8^ To address this, we developed a method to create intermediate progenitor cells, termed oligodendrogenically biased NPCs (oNPCs), which are biased toward differentiating into more oligodendrocytes while still preserving their tripotency.^9-11^

Following oNPC transplantation into a thoracic rodent model of SCI, the grafted cells demonstrated promising outcomes, such as differentiating into a higher proportion of oligodendrocytes, promoting remyelination, enhancing axonal sparing, and facilitating functional recovery.^10,11^ However, it is crucial to note that thoracic injuries represent only 32% of the patient population, while the majority of patients (approximately 60%) suffer from cervical injuries.^12^ Given that the thoracic and cervical spinal cord regions are anatomically, structurally, and functionally distinct, the outcomes observed in thoracic models may not be directly applicable to cervical cases. Each spinal cord region plays specific roles in controlling movement, sensation, and autonomic functions, with the cervical spinal cord responsible for motor neuron pools that control muscles of the head, neck, shoulders, arms, and hands, while the thoracic spinal cord controls motor neuron pools for the trunk muscles. Similarly, sensory neurons and interneurons also exhibit differences in their distribution and organization across distinct areas of the spinal cord. Anatomically, the cervical spinal cord has a greater white matter area, more myelinated axons, and increased myelin thickness compared to the thoracic spinal cord.^13-15^ Consequently, cell therapies may have different implications and success rates in the thoracic versus the cervical spinal cord. For example, white matter regeneration and remyelination may be a more relevant therapeutic target in cervical SCI, due to the enrichment of myelin in this region. As such, in the present study, we aimed to test the therapeutic capacity of oNPCs in promoting regeneration in a clinically relevant bilateral contusive-compressive model of cervical SCI, which recapitulates the pathophysiology that is seen in human cervical injuries.^16^

## Methods

### hiPSC Culture and Differentiation of hiPSCs to oNPCs

The hiPSC line BC1 was obtained from the Centre for Commercialization of Regenerative Medicine (CCRM) in Toronto. The hiPSCs were cultured in mTeSR1 medium (STEMCELL Technologies, Vancouver, ON) and were subjected to several passages to allow them to adapt to the culture conditions. The hiPSCs were then differentiated to NPCs using dual SMAD inhibition in monolayer culture^17^ with some modifications. Partially differentiated colonies were manually removed before differentiation. At the start of differentiation (day 0), hiPSCs were dissociated to single cells and replated as a monolayer on Matrigel (Corning) with a density of 20 000 cells/cm^2^ in mTeSR1 media. After the cells reached 90% confluence, media was gradually changed over 2 days to neural induction media consisting of a 1:1 ratio of DMEM:F12 media supplemented with B27, N2, FGF (10 ng/mL), 10 μM TGFβ inhibitor (SB431542), 200 ng/mL Noggin, and 3 μM GSK3β inhibitor (CHIR99021). After 7 days in culture, the neural rosettes were manually isolated and plated as single cells on poly-l-lysine (PLL)/Laminin-coated dishes in NPC expansion media (NEM) consisting of neurobasal media supplemented with B27, N2, FGF (10 ng/mL), and EGF (20 ng/mL) for 2 passages. The resulting cells were then cultured in NEM as single cells on Ultra-Low adherent dishes (Corning) at a density of 10 000 cells/mL to form primary neurospheres. After 5 days in culture, each individual clonal neurosphere was separately plated in a well of a PLL/Laminin-coated 24-well plate to proliferate. The steps were then repeated to get the secondary clonal neurospheres. For expansion of the culture, secondary clonal neurospheres were cultured in NEM on PLL/Laminin. During the period of induction, which took over 2 weeks, the cells progressed through the neural rosette and neurosphere stages. The NPCs were subsequently infected with a lentiviral construct expressing GFP in order to allow for the visualization of the transplanted cells. Next, the NPCs were biased toward oNPCs, as described previously with a number of minor modifications.^9^ Briefly, the NPCs were caudalized by culturing them on growth factor reduced Matrigel in DMEM/F12, supplemented with 0.1 μM retinoic acid (RA), B27 supplement, N2 supplement, and EGF (20 ng/mL) for 3 days. Cells underwent ventralization by treatment with 1 μM sonic hedgehog (Shh) agonist Purmorphamine for 5 days. EGF was replaced by FGF-2 (10 ng/mL) from the media for 3 days followed by the addition of 20 ng/mL PDGF-AA for 14 days. The resulting cells were maintained on Laminin-coated dishes in DMEM/F12, B27-A, N1 supplement, PDGF-AA (20 ng/mL), and FGF-2 (20 ng/mL) for 3 more passages prior to transplantation. During passaging, 10 μM Rock inhibitor (Y-27632) was added on day 1.

### Immunocytochemistry of the Cultured Cells

The oNPCs were treated with differentiation media without fibroblast growth factor 2 (FGF2)/epidermal growth factor (EGF) and supplemented with 0.1% fetal bovine serum on coverslips coated with 10 μg/mL laminin for 20 days. The cells were then fixed with paraformaldehyde in PBS (4%) and sucrose (40%) for 20 minutes. The fixed cells were then treated with Triton X-100 (0.1%) and sodium citrate in PBS (0.1%) for 5 minutes and blocked in BSA (5%) at room temperature for 1 hour. The cells were incubated with the appropriate primary antibodies in blocking buffer overnight at 4 °C. The following primary antibodies and concentrations were used for fluorescent staining: GFAP (1:1000, AB5804, Millipore), Tuj1 (1:500, MMP-435P-100, Covance), and O4 (1:500, MAB345, Millipore). For O4 staining, the treatment with Triton X-100 was not done. The cells were subjected to a series of washing steps the following day and subsequently incubated for an hour with fluorophore-conjugated secondary antibodies and 4ʹ,6-diamidino-2-phenylindole (DAPI, 1:1000, D1306, Invitrogen). In order to quantify the percent differentiation, a total of 3 wells were stained for each mature cell marker, and the number of positive cells was counted in 5 different areas in each well. The percent differentiation was determined relative to the total number of DAPI+ cells in each area.

### Reverse Transcription-Quantitative Polymerase Chain Reaction (RT-qPCR)

Small-molecule-treated oNPCs and NPCs were collected, and RNA was isolated using the Total RNA Purification Kit (17200, Norgen Biotek). cDNA was then synthesized using the SuperScript First-Strand Synthesis System for RT-PCR (11904018, Thermo Fisher Scientific). RT-PCR was performed on the GeneAmp PCR System 9700 with the appropriate TaqMan probes. The reaction was set to 94 °C for 2 minutes, followed by 35 cycles switching between 94 °C for 30 seconds, 58 °C for 30 seconds, and 72 °C for 60 seconds. The GAPDH housekeeping gene and an NPC control sample were used for normalization.

## Rat Model of Cervical Spinal Cord Injury

All animal procedures were carried out in accordance with the University Health Network (UHN) Animal Care Facility rules (Toronto, ON) and in accordance with the policies established by the Canadian Council of Animal Care’s guide to the care and use of experimental animals. Animals were subjected to a contusion-compression cervical SCI using a modified aneurysm clip, which has been extensively characterized by our laboratory. Briefly, adult female athymic Rowett Nude (RNU; NIH-Foxn1rnu) rats (180-200 g; Charles River, Montreal, Canada) were deeply anesthetized using 4% isoflurane and were sedated for the remainder of the surgery under 2% isoflurane. Animals received a 2-level laminectomy of the cervical vertebral segments C6-C7. A modified clip calibrated to a closing force of 21.5 g was applied extradurally to the cord for 1 minute and then removed. Sham-operated rats received a laminectomy without the clip compression injury. A small square of Surgifoam (Ethicon Endo-Surgery, Inc., Cincinnati, OH) was placed over the injury site, and the overlaying muscle and skin were sutured. Postoperatively, animals were treated with analgesics (0.05 mg/kg buprenorphine) and saline (0.9%; 5 mL) to prevent dehydration. Animals were housed individually in standard rat cages with absorbent bedding at a temperature of 27 °C for recovery. Injured animals had their bladders manually expressed 3 times daily until natural bladder function returned.

### Intraspinal Transplantation

Injured animals were randomly divided into 2 groups to receive either oNPC transplantation (*n* = 15) or vehicle injection as a control (*n* = 15) on day 14 after injury. Under isoflurane (1%–2%) and a 1:1 mixture of O_2_/N_2_O, rats were placed in a stereotactic frame and administered 0.05 mg/kg buprenorphine as well as 10 mL saline, and injuries were carefully reexposed. Dissociated oNPCs suspended in artificial cerebrospinal fluid at 50 000 cells/μL were injected intraspinally at 4 sites 0.5-1.0 mm bilateral to the midline and 2 mm rostral and caudal, 2.0 μL each, for a total of 4 × 10^5^ cells per animal. Injections were delivered at 0.6 μL/minute, held for 2 minutes, and retracted over 2 additional minutes using a Hamilton syringe and a stereotaxic injection system (System UMP3 with Micro4, World Precision Instruments, Sarasota, FL). The control animals also received the same number of injections to the spinal cord with only artificial cerebrospinal fluid.

### Tissue Processing

At 10 weeks postinjury, the rats were deeply anesthetized with isofluorane and transcardially perfused with 180 mL of ice-cold phosphate-buffered saline (PBS) and 180 mL of 4% paraformaldehyde in PBS. Following collection, spinal cord tissues were fixed for 5 hours and then cryoprotected in 30% sucrose in PBS for 24 hours. Spinal cord segments were embedded, frozen, and stored at −80 °C. A section centered around the site of injury was sectioned using a cryostat at a thickness of 30 µm.

### Tissue Immunohistochemistry and Quantification

To perform immunohistochemistry and quantification, tissue sections were first immersed in PBS for 5 minutes. This was followed by a 1-hour incubation at room temperature in blocking buffer, containing 1% bovine serum albumin (BSA), 5% skim milk, and 0.3% Triton-X 100 in PBS. After removing the blocking solution, primary antibodies were applied and incubated overnight at 4 °C, followed by three 10-minute PBS washes. The following primary antibodies and concentrations were used for fluorescent staining: APC (1:40, OP80, Millipore), Olig2 (1:500, AB9610, Millipore), GFAP (1:1000, AB5804, Millipore), MBP (1:100, AB7349, Abcam), NeuN (1:1000, MAB377, Millipore), Tuj1 (1:500, MMP-435P-100, Covance), Ki67 (1:500, AB16667, Abcam), and Nestin (1:200, MAB5326, Millipore). The slides were then rinsed and incubated with Alexa Fluor 568 fluorescent secondary antibodies at a 1:500 dilution for 2 hours at room temperature. The total number of cells was assessed by counting the number of GFP+/DAPI+ cells on serial sections and using the Cavalieri tool in the Stereo Investigator software to estimate the total number of cells (*n* = 5).

For the staining of NF200 (1:500, N0142, Sigma), a secondary antibody conjugated with horseradish peroxidase (HRP; Invitrogen; 1:200) and 3,3ʹ-diaminobenzidine (DAB) staining (Vector Laboratories) were used. The NF200+ area was measured at 0 and 3.00 mm rostral and caudal to the injury epicenter (*n* = 5 per group). Five regions on axial sections of the spinal cord were imaged using a Leica fluorescence microscope at 20× magnification, with identical exposure time, gain, and offset values. In ImageJ, randomized NF200-stained sections were batch converted to 8-bit grayscale, and a thresholding procedure was applied to all images to discern NF200+ neurofilaments.

To measure the area of GFAP staining, the entire horizontal section was imaged at 20× magnification using a Leica fluorescence microscope with identical exposure time, gain, and offset values at 0, 960, 1920, and 2880 μm rostral and caudal to the epicenter. Uninjured control tissue was used for normalization in the linear histogrammatic range. In ImageJ, GFAP-stained images were batch converted to 8-bit grayscale, and a 3-step threshold procedure was uniformly applied to all images to accurately detect GFAP intensity (*n* = 5 per group).

To count the number of NeuN+ and ChAT+ (1:100, AB143, Millipore) cells (*n* = 5 per group), a total of 7 sections rostral and caudal to the injury epicenter were used. The region of interest was the ventral horn, defined anteriorly and laterally by the grey matter-white matter boundary, medially by the ventral horn-grey commissure junction, and dorsally by a line extending from the ventral edge of the grey commissure. Sections were imaged on a Zeiss LSM-510 confocal microscope at 20× magnification. The number of NeuN+/DAPI+ and ChAT+/NeuN+ neurons in the region of interest were manually counted using the “Cell Counter” plugin in ImageJ software (NIH).

### Immune-Electron Microscopy

Immune-electron microscopy was performed as previously described.^18^ Briefly, frozen sections were blocked with 5% blockace and 0.01% saponin in 0.1 M phosphate buffer and subsequently incubated with an anti-GFP mouse monoclonal antibody and a nanogold-conjugated anti-mouse IgG secondary antibody (1:100, Invitrogen). Sections were fixed with 2.5% glutaraldehyde, postfixed with 0.5% OsO_4_ and embedded into epon. Ultrathin sections (70 nm thick) were prepared, stained with uranyl acetate and lead citrate, and observed under a transmission electron microscope (TEM, JEOL 1400plus).

### Tissue Histomorphometric Analysis

Spinal cord sections were stained with Luxol Fast Blue (LFB) and Hematoxylin & Eosin (H&E). LFB is a myelin-selective stain, while H&E are stains for all cell nuclei and cytoplasmic proteins, respectively. Slides were retrieved from −80 °C, baked at 56 °C for 15 minutes, and washed using various mediums in preparation for the overnight LFB stain at 56 °C. On the following day, tissues were retrieved from the oven and processed through various washing steps for H&E staining. Afterward, tissues were sequentially dehydrated with increasing concentrations of alcohol and xylene, and ultimately cover slipped. A blinded investigator performed LFB and H&E analyses on tissue ±2400 µm centered at the injury epicenter. Unbiased measurements were made using Cavalieri volume probe from Stereo Investigator (MBF, Bioscience, Wilson, VT; http://www.mbfbioscience.com/) for area and volume estimations of the preserved grey and white matter, the cavitation, and the lesional tissue.^19^ Briefly, the lesional tissue was identified by microcystic degeneration, astrogliosis, chronic inflammation, and fibrovascular changes. Calculations and analyses were done for tissue sections every 240 µm (*n* = 5 per group).

### In Vivo Very High-Resolution Ultrasound Imaging for Cavitation Analysis

Very high-resolution ultrasound (VHRUS) imaging was performed at the endpoint prior to sacrifice as previously described.^20^ Under isoflurane anesthesia, animals (*n* = 5 per group) were placed within a custom-made stabilization frame on the imaging platform (Vevo imaging station, Visualsonics, Toronto, Canada). The injury was reexposed through a midline incision and ultrasound gel (Scanning Gel, Medi-Inn, Canada) was placed on the dura mater. The spinal cord was scanned with the VHRUS probe (44 MHz, Vevo 770, Visualsonics, Toronto, Canada) in 3Dmode.^21^

### Behavioral Assessment

All neurobehavioral assessments were performed and analyzed by examiners blinded to the treatment groups. Grip strength, Basso, Beattie and Bresnahan (BBB) scale, and inclined plane tests were performed once per week for 10 weeks after injury (*n* = 12 in vehicle group, 12 rats in oNPC group, 8 rats in sham group). Forelimbs were specifically tested with a grip strength meter (GSM) for composite paw strength.^19,22^ To evaluate grip strength, animals’ hindlimbs and lower abdomen were drawn backward at a consistent speed within reach of a metal rung connected to a GSM apparatus. Animals grasp the rung reflexively, and a force gauge measures the maximal force achieved when grip is broken. Scores from 5 successful grasps were averaged. Hindlimb function was tested using the 21-point open-field BBB locomotor scale. To assess whole-body limb and trunk motor function, the inclined plane test was performed, as described previously.^19,22^ Rats were placed on a horizontal plane that was pivoted to increase the level of incline from 15° to 90° at increments of 2.5°. The rats were placed with their body axis parallel to the axis of incline for 5 attempts at each incline level. They were required to maintain their body position on the raised plane for 5 seconds for 3 or more of the 5 attempts in order to move on to the next incline level.

The CatWalk automated gait analysis system (Noldus, Wageningen, The Netherlands), was used to evaluate the precise forelimb and hindlimb movements at 10 weeks after SCI (*n* = 12 in the vehicle group, 12 in the oNPC group, 8 in the sham group). Each rat was placed in front of the start zone of the CatWalk runway, and footprints of the animal crossing a glass walkway were recorded by the video camera positioned below. For data collection, 2 uninterrupted runs per animal were performed. The values for every paw were taken, and averages between left and right paws were used for analysis. All animals could walk along the walkway for the analysis. In the present study, we analyzed the following data with the CatWalk program, version 10.5 (Noldus): (1) fore- and hindlimb stride length: distance between at least 3 consecutive paw placements, and (2) fore- and hindlimb swing speed: the speed of the paw during the swing phase (the duration of no paw contact with the glass plate during a step cycle).

Thermal allodynia was evaluated once per week for 8 weeks after transplantation (*n* = 11 in the vehicle group, 12 in the oNPC group, and 4 in the sham group). In brief, thermal allodynia was assessed by the tail-flick latency time in response to a light beam using a Tail Flick Analgesia Meter Apparatus (Model 336T6; IITC Life Science). The latency of the rat to remove its tail from the heat was recorded. In addition, mechanical allodynia was investigated at 8 and 10 weeks following injury (*n* = 8 in the vehicle group, 8 in the oNPC group, and 5 in the sham group). The rats were allowed to acclimate to the testing environment for 30 minutes before testing. Mechanical allodynia was assessed using a 4 g von Frey filament (Semmes-Weinstein monofilaments; Stoelting). The rat enclosure had a metal mesh floor that allowed for the application of the filament to the plantar surface of the fore- and hindpaws. Ten trials were carried out during the testing week with a minimum of 15 minutes between each trial. Paw withdrawal after stimulus was counted as a response.

### Statistical Analyses

With close animal care, total exclusion and mortality were 15% from all causes. Humane euthanasia endpoints and exclusion criteria were determined a priori prior to the experiments and assessed blindly. A total of 4 animals were excluded from statistical analysis due to skin lesions. The following humane end points were determined a priori for exclusion and/or euthanasia: weight loss exceeding 20% of preoperative weight, not eating or drinking for more than 24 hours, anorexia and chronic urinary tract infection, or hematuria that did not respond to antibiotics. Autophagia of the paws was treated with meloxicam and buprenorphine, but if ineffective, was followed by euthanasia. Exclusion and mortality were not statistically different between groups. Results are stated as mean ± SEM, and *P*-values < .05 were considered significant. Assumptions of normality and homogeneity of variances were evaluated using Shapiro Wilk’s test and Fligner-Killeen’s test, respectively. Immunohistological data and the VHRUS data were analyzed using Student’s *t*-tests. Histomorphometric and behavior data were analyzed using 2-way repeated-measures analysis of variance (ANOVA) with a Tukey posthoc test, or a one-way ANOVA with a Tukey posthoc test. Statistical analyses were conducted, and graphical presentations were prepared in R (version 4.1.2) or Prism 7 (GraphPad Software, San Diego, CA).

## Results

### Differentiation of oNPCs from hiPSC-NPCs

**Figure 1.**
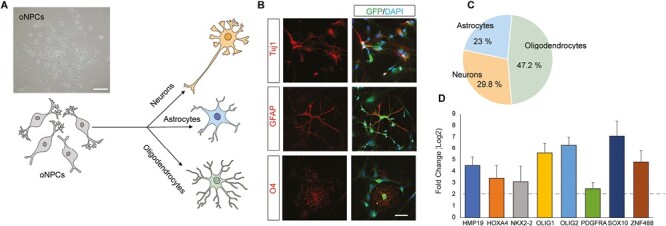
Generation and characterization of oNPCs. (**A**) Micrograph showing the monopolar and bipolar morphology of the oNPCs. Scale bar = 40 µm. (**B** and **C**) Immunofluorescence analysis of neuronal (Tuj1), astrocytic (GFAP), and oligodendroglial (O4) markers after 20 days of differentiation demonstrates the trilineage potential of the oNPCs. Scale bar = 10 µm. (**D**) RT-qPCR analysis comparing the expression profile of oNPCs to unbiased NPCs. Relative gene expression was determined by the 2^−ΔΔCT^ method and fold change values are relative to the mean gene expression in the housekeeping gene, GAPDH. (*n* = 3 in each analysis).

oNPCs were generated from hiPSC-NPCs based on a protocol previously established by our lab in which the cells are treated with a series of small molecules that mimic oligodendroglial development.^9^ The resulting cells demonstrated typical elongated monopolar and bipolar morphologies (Fig. 1A). To determine the differentiation potential of the oNPCs, cells were cultured in differentiation conditions. Specifically, the oNPCs were cultured with the addition of 0.1% fetal bovine serum for 20 days. After this period, the morphological changes in the cells were accompanied by the expression of markers characteristic of astrocytes (GFAP+; 23 ± 3.7%), neurons (Tuj1+; 29.8 ± 3.1%), and oligodendrocytes (O4+; 47.2 ± 5.1%), as demonstrated by immunocytochemistry (Fig. 1B and 1C). Next, mRNA expression levels of several genes that have been shown to be involved in the differentiation of oligodendrocytes were analyzed using RT-qPCR (Fig. 1D). The expression levels of *OLIG1*, *OLIG2*, and *SOX10* were much higher in oNPCs in comparison to unbiased NPCs (Fig. 1D).

### Engrafted oNPCs Migrate Rostrocaudal to the Lesion and Preferentially Differentiate into Oligodendrocytes

**Figure 2.**
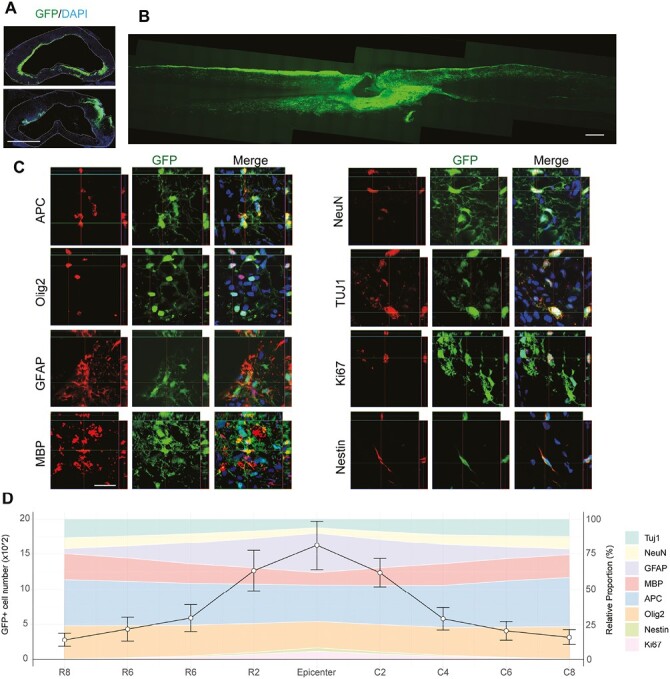
oNPCs survive, integrate, and differentiate in spinal cord tissue following SCI. (**A**) Representative axial images display the lesion epicenter in the spinal cord. Scale bar = 1000 µm. (**B**) A longitudinal section at the midline demonstrates that oNPCs survive, disperse within the tissue, and migrate around lesion sites. Scale bar = 1000 µm. (**C**) Representative confocal images, which include orthogonal views for both the XZ (vertical plane along the depth) and YZ (vertical plane perpendicular to the XZ plane) planes, show GFP+ transplanted cells differentiating and expressing markers for mature oligodendrocytes (APC), immature oligodendrocytes (Olig2), myelinating oligodendrocytes (MBP), astrocytes (GFAP), and neurons (TUJ1 and NeuN). Ki67 and Nestin staining indicate proliferative and undifferentiated cells, respectively. Scale bar = 20 µm. (**D**) The left axis displays a quantitative analysis of the number of GFP+ grafted cells, which have been measured at 2 mm intervals from the epicenter of the spinal cord. The intervals on the rostral side are labeled from R2 to R8, while those on the caudal side range from C2 to C8. The right axis corresponds to the stacked area graph and presents the relative proportion of each stain shown in the panel (*n* = 5).

To assess the therapeutic potential of the oNPCs for cervical SCI, we transplanted these cells into athymic RNU *Foxn1*−/− rats during the subacute phase, at 14 days postinjury. Eight weeks following transplantation, we quantified the number of GFP+ human cells in the rat spinal cord tissue using Cavalieri’s principle for volume estimation. We observed an average of 51 ± 8.2 × 10^3^ (mean ± SEM) cells within a 16 mm distance on either side of the injury epicenter. The engrafted oNPCs exhibited extensive migration, spanning up to 8 mm in both rostral and caudal directions from the lesion site (Fig. 2A and 2B).

The grafted GFP+ cells were primarily concentrated around the injury epicenter. Immunostaining for proliferation and neural markers indicated that most oNPCs had differentiated into neurons, astrocytes, and oligodendrocytes, thus, confirming their tripotency. A limited number of Nestin+ grafted cells (0.31 ± 0.14%) remained, with the majority situated near the epicenter. We detected proliferative GFP+ cells expressing Ki67 (2.67 ± 0.48% of GFP+ cells) within a distance of 4 mm rostral and caudal to the epicenter (Fig. 2C and 2D).

The GFAP+ astrocytes were predominantly located toward the epicenter, comprising 18.08 ± 2.27% of all GFP+ cells. Intriguingly, oligodendrocytes at varying developmental stages were more concentrated away from the epicenter. Olig2, expressed in immature oligodendrocytes, was present in 37.98 ± 7.99% of the total GFP+ cells. APC, expressed in late-stage immature oligodendrocytes and mature oligodendrocytes, was identified in 53.23 ± 6.24% of the total grafted human GFP+ cells. Moreover, MBP-expressing GFP+ cells, representing mature, myelinating oligodendrocytes, constituted 23.35 ± 8.38% of the total GFP+ cells (Fig. 2C and 2D). The distribution pattern of neurons along the rostral-caudal axis of the spinal cord resembled that of oligodendrocytes. GFP+ cells expressing Tuj1 (class III β-tubulin) denoted immature and newly differentiated neurons in their early developmental stages, accounting for 14.62 ± 1.67% of the total GFP+ cells. NeuN (Fox3)-expressing GFP+ cells, indicative of mature neurons in their later stages of development, comprised 5.51 ± 0.33% of the total GFP+ cells. Overall, we noted that the proportion of oligodendroglial and neuronal differentiation increased with distance from the injury, while astrocyte differentiation was higher at the injury epicenter and lower at remote sites.(Fig. 2C and 2D).

We performed a comprehensive examination of RNU rats up to 8 months posttransplantation to ensure the safety of the transplanted cells. We did not observe any evidence of tumor tissue in the spinal cord at 8 months posttransplantation (Supplementary Fig. S1). These results suggest that the risk of tumorigenicity is low in the oNPCs within the context of the RNU rat model of cervical SCI.

### Transplanted oNPCs Contributed to Remyelination in the Injured Spinal Cord Tissue

**Figure 3.**
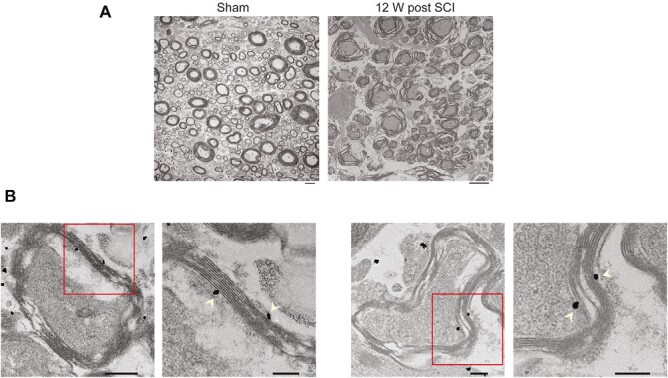
Transplanted oNPCs contribute to remyelination following SCI. (**A**) Electron micrographs of the dorsal column white matter in the cervical spinal cord demonstrated compromised structural integrity of the myelin sheath, with massive disruptions and disassembly surrounding axonal fibers 12 weeks following SCI. Scale bars = 2 µm. (**B**) Representative images of electron micrographs with black dots (nanogold particles) representing the GFP+ transplanted cells. The grafted cells are in close proximity to the thin lamellar structures, indicating remyelination. The sections highlighted in the red box can be seen enlarged in the panels to the right. Scale bars in unenlarged panels = 500 nm, enlarged panels = 200 nm.

We analyzed the impact of cervical SCI on the ultrastructure of the surrounding myelin sheath using electron microscopy. Our findings revealed significant disruptions to the lamellar myelin structures, indicative of myelin damage. We observed fragmented and disintegrated myelin sheath, disorganized and irregular myelin lamellae, and the presence of irregular shapes and debris. In comparison to the sham controls, injured spinal cords exhibited thinner and less compact myelin sheath, further highlighting the detrimental effects of SCI on myelin structure (Fig. 3A). These observations are consistent with previous reports showing that compressive forces associated with SCI can cause oligodendrocyte necrosis and necroptosis, leading to myelin degradation.^2,5^

To assess the potential of transplanted cells for remyelination, we performed immunogold electron microscopy using an anti-GFP antibody to identify grafted cells in sections obtained from grafted RNU rats at 10 weeks postinjury. We observed multilayered myelination in close proximity to the nanogold-labeled cells (Fig. 3B). This result suggests that the transplanted cells may contribute to the process of remyelination following SCI.

### oNPC Transplantation Enhances Tissue Preservation After SCI

**Figure 4.**
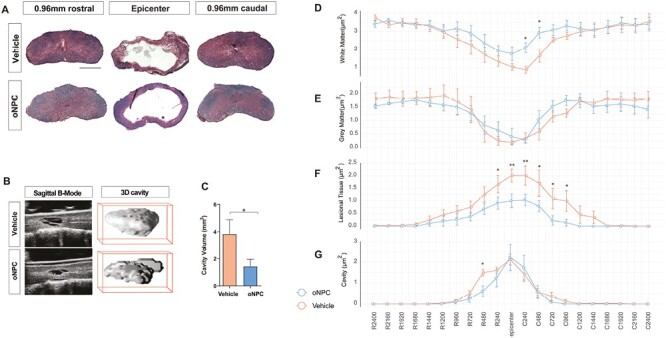
Histomorphometric analysis using LFB and H&E staining. (**A**) Representative images of the spinal cord at the lesion epicenter as well as 0.96 mm rostral and caudal area for a vehicle and an oNPC-treated animal. Scale bar = 1000 µm. (**B**) High-resolution ultrasound (VHRUS) images of the spinal cord in 2D, and the cavity in 3D. (**C**) Quantitative analysis of the cavity volume. Quantitative analysis of the area of: (**D**) white matter, (**E**) grey matter, (**F**) lesional tissue, and (**G**) cavity at different rostral and caudal interval distances (µm) from the injury epicenter. *P < .05 and **P < .01. (*n* = 5 per group).

We subsequently conducted a histomorphometric analysis of the tissue following cell transplantation in comparison to vehicle treatment to assess the extent of preserved tissue, cavity size, and lesional tissue. To achieve this, we performed Luxol Fast Blue (LFB) and Hematoxylin & Eosin (H&E) staining on tissue sections (Fig. 4A). Analysis of LFB and H&E-stained sections revealed that oNPC-treated animals had greater white matter areas than vehicle-treated animals at both 240 µm and 480 µm caudal to the epicenter (Fig. 4D). While grey matter areas were not significantly different among the groups (Fig. 4E), the lesional tissue sizes in the oNPC group were significantly smaller compared to the vehicle group (Figure 4F). In addition, the cavity sizes were smaller in the oNPC group rostral to the epicenter (Figure 4G).

In addition to LFB and H&E staining, we used high-resolution ultrasound (VHRUS) imaging^20,21^ to obtain a more accurate analysis of cavity size without tissue shrinkage. VHRUS imaging can generate planar full-depth images and 3D reconstruction volumes of cavitation in situ, helping to overcome tissue shrinkage difficulties that occur during routine histological processing.^20^ The oNPC-treated animals exhibited significantly smaller cavities compared to the vehicle-treated animals (vehicle: 3.84 ± 1.05 mm³, oNPCs: 1.43 ± 0.52 mm³; *P* < .05, one-way *t*-test, Fig. 4B and 4C).

### oNPC Engraftment Reduces Astrogliosis and Promotes Axonal Preservation

**Figure 5.**
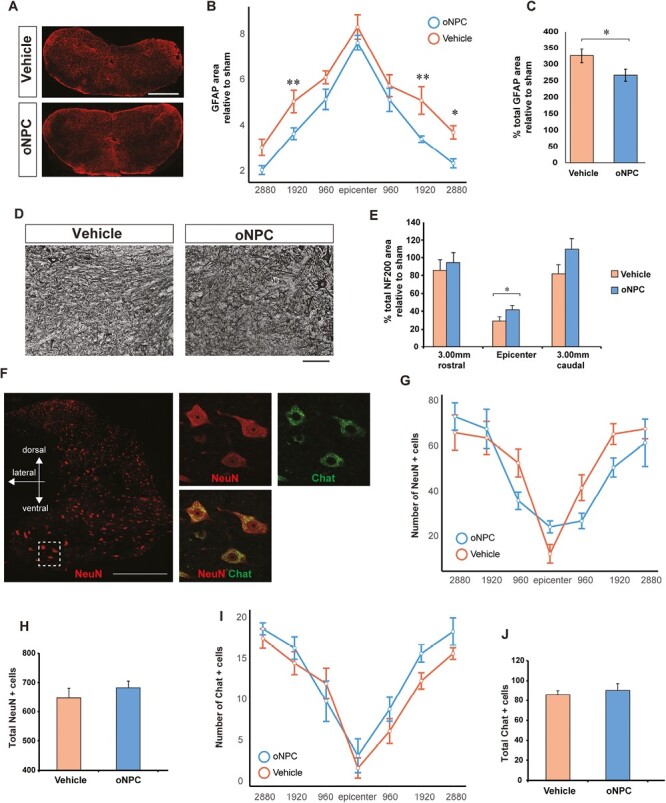
Astrogliosis and axonal preservation following transplantation. (**A**) Representative images of GFAP staining at 960 μm rostral to the epicenter. Scale bar = 1000 µm. (**B** and **C**) Quantitative analysis of the GFAP area at varying distances from the epicenter (µm) and the percentage of total GFAP area relative to sham. (**D**) Representative images of NF200 in grey matter at the epicenter. Scale bar = 100 µm. (**E**) Quantitative analysis of the percentage of the NF200 area at the epicenter and 3 mm rostral and caudal from the epicenter relative to the sham. (**F**). Representative images of NeuN+ and/or ChAT+ neurons in the grey matter. Scale bar = 500 µm. (**G** and **H**) Quantitative analysis of the number of NeuN+ cells at varying distances from the epicenter (µm) and the total number of NeuN+ cells. (**I** and **J**) Quantitative analysis of the number of ChAT+ cells at varying distances from the epicenter (µm) and the total number of ChAT+ cells. Axial sections in (A), (D), and (F). *P < .05 and **P < .01. (*n* = 5 per group).

After assessing spinal cord preservation, further histological analysis of the lesion was performed. Transplanted cells showed engraftment within the reactive astrocyte scar area at the lesion epicenter. As our previous work suggested that cell transplantation contributes to a reduction in the glial scar,^19,22^ we investigated whether cell transplantation could modulate formation of the glial scar by immunostaining for GFAP (Fig. 5A). At 10 weeks after injury, the % total GFAP area in the oNPC group (oNPCs: 266.84 ± 18.3%) showed a significant reduction compared to vehicle (326.25 ± 21.5%; Fig. 5B and 5C). Thus, oNPC transplantation suppressed the progression of astrocyte reactivity. Given that the glial scar is known to inhibit axonal extension by forming a chemical and physical barrier, we next investigated whether the observed suppression of astrogliosis may have an effect on neural regeneration across the lesion site by immunostaining for NF200. The NF200+ neuronal fibers were significantly more abundant in the oNPC group (41.37 ± 4.8%) compared with the vehicle group (29.00 ± 4.90%) in the epicenter of the lesion (*P* < .05) (Fig. 5D and 5E).

### The Effect of oNPC Transplantation on the Survival of Endogenous Neurons

The local neuronal network plays a crucial role in facilitating the recovery of forelimb and hindlimb neurobehavioral function following cervical SCI. Therefore, neuronal preservation is an important factor that influences neural signaling. To investigate the number of preserved endogenous neurons and motor neurons, we assessed NeuN+ and ChAT+ cells spanning a distance 2880 µm rostral to 2880 µm caudal to the injury epicenter, at 960 µm intervals (Fig. 5F). The endogenous neurons were distinguished from the graft-derived neurons by a lack of GFP staining. The number of endogenous NeuN+ neurons in the oNPC transplanted rats was not significantly different from the vehicle group (Fig. 5G and 5H). In addition, we did not find a significant difference in the number of ChAT+ motor neurons between the oNPC transplanted rats and the vehicle group (Fig. 5I and 5J).

### Transplantation of oNPCs Improves Functional Recovery

**Figure 6.**
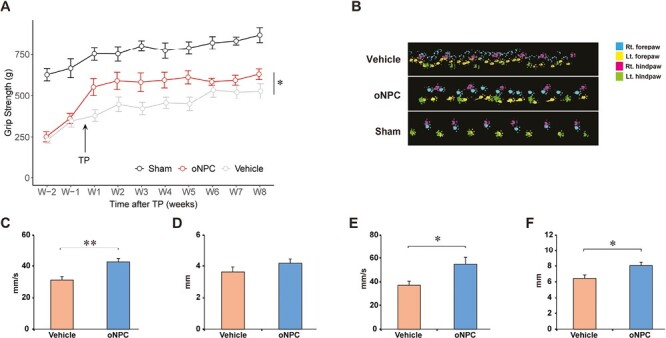
Functional analysis following cell transplantation. (**A**) Time course of grip strength recovery. The oNPC group had significantly better grip strength than the vehicle group. Means ± SEM, repeated-measures 2-way ANOVA, Tukey’s posthoc, *P < .05. (**B**) Representative images of gait analysis with the CatWalk system 10 weeks after SCI. Quantification of gait analysis in the forelimbs and hindlimbs: (**C**) swing speed in the forelimbs, (**D**) stride length in the forelimbs, (**E**) swing speed in the hindlimbs, and (**F**) stride length in the hindlimbs. Student’s *t*-test, *P < .05; **P < .01. (*n* = 12 in the vehicle group, 12 in the oNPC group, and 8 in the sham group).

Given the histopathological improvements, we investigated whether these histological changes were associated with better functional recovery. To assess functional recovery, we first examined forelimb grip strength. All animals demonstrated gradual neurological improvement over time. The oNPC transplanted rats showed significantly better function compared to the vehicle-treated rats (Fig. 6A).

Furthermore, we quantified gait and locomotor function at 10 weeks after injury using the CatWalk gait analysis system (Fig. 6B). With respect to forelimb function, statistically significant differences were seen between the oNPC group (42.60 ± 2.40 mm/s; *P* < .01) compared to the vehicle group (31.14 ± 2.32 mm/s; Fig. 6C). oNPC transplantation did not result in significant improvements in forelimb stride length compared to vehicle (Fig. 6D). Hindlimb functional recovery was observed in the oNPC group compared to the vehicle control group in both swing speed (vehicle: 37.08 ± 3.41 mm/s vs. oNPCs: 54.92 ± 5.89 mm/s; *P < .*05; Fig. 6E) and stride length (vehicle: 6.43 ± 0.47 mm vs. oNPCs: 8.06 ± 0.44 mm; *P* < .05, Fig. 6F).

The BBB scale and inclined plane test were performed as well but did not detect a difference among the groups (Supplementary Fig. S2A and S2B). This is consistent with our previous finding that BBB is not a suitable behavioral test for the assessment of recovery in cervical models of SCI.^19^

Increased neuropathic pain is a potential concern after cell-based treatments.^23^ Consequently, we assessed thermal and mechanical allodynia. The latency times for the rats to remove their tails from a focal heat source (Supplementary Fig. S2C) were not significantly different at any time point among the groups. In addition, there was no significant difference in their responses to the forelimb and hindlimb application of the von Frey filament at 8 and 10 weeks after injury (Supplementary Fig. S2D and S2E). These data indicate that oNPC transplantation therapy does not increase neuropathic pain in our model.

## Discussion

Cell transplantation represents a plausible strategy to harness neuroregeneration following SCI. Although different cells have been transplanted for SCI with varying degrees of success, the optimal cell type for recovery has yet to be identified. Moreover, the majority of preclinical studies have focused on cell transplantation in thoracic models of SCI, as these models have been more readily available historically.^24,25^ The present study addresses a key knowledge gap by assessing the therapeutic relevance of oNPCs in a clinically relevant model of cervical SCI.

In the present study, we found that the oNPCs had an enhanced oligodendendroglial differentiation capacity in vitro as well as in vivo and contributed to remyelination, thus addressing important consequences of SCI. Notably, the injury site is characterized by central cavitation and a subpial rim of surviving small-diameter axons with varying degrees of demyelination.^26,27^ Our group and others have shown that axonal demyelination at the injury site results in axonal dysfunction and functional neurological deficits.^26-28^ Of note, relatively small changes in conduction can substantially influence functional recovery.^29^ As such, our electron microscopy results suggest that newly formed multilayer myelin may contribute to the functional benefits that were seen in our behavioral analyses.

Our differentiation analyses also indicated that the oNPCs were capable of differentiating into neurons. This is an important property of the cells, as in addition to oligodendrocyte death, SCI is followed by extensive neuronal loss which warrants the replacement of neurons as well. Notably, exogenous neurons have been shown to make synapses with endogenous neurons and to improve neural circuit conduction.^30,31^ Previous studies have also demonstrated that there is an association between motor function recovery and neuronal progenitor transplantation.^30–32^ For example, Abematsu et al. reported that the neuronal differentiation of transplanted NPCs significantly enhanced functional recovery following thoracic SCI. Meanwhile, the ablation of these transplant-derived cells abolished hindlimb functional recovery, ultimately suggesting that the transplanted neuronal cells directly contribute to functional recovery in the hindlimb.^32^ Therefore, the neuronal differentiation potential of the oNPCs makes these cells favorable over traditional OPCs, which cannot replace lost neurons.

In order to determine the effect of lesion proximity on oNPC differentiation, we also quantified oligodendrocyte, neuron, and astrocyte differentiation at the injury epicenter, as well as rostral and caudal from the injury. The oNPCs consistently gave rise to a greater proportion of oligodendrocytes, except at the injury epicenter, where astrocyte differentiation was higher. This finding is not surprising, as the molecular profile across the length of the cord is not homogenous post-SCI. Specifically, SCI induces differential gene expression patterns and signaling gradients at varying distances from the injury site and across different areas of the glial scar.^33,34^ This may ultimately exert distinct effects on the grafted oNPCs that have migrated to the injury core versus those that migrated away from the injury.

In addition to contributing to maladaptive signaling cascades, the glial scar, which forms in the chronic stages following SCI and consists of reactive astrocytes and chondroitin sulfate proteoglycans (CSPGs), introduces a significant physical barrier that restricts axon regeneration and remyelination.^35,36^ Several research efforts have been directed toward reducing the glial scar through the injection of chondroitinase ABC (ChABC), which is an enzyme that degrades CSPGs.^36^ In our previous oNPC study in thoracic SCI, we combined oNPC transplantation with ChABC administration and found that this combination contributed to the reduction of the glial scar.^11^ However, it has been reported that stem cells alone can attenuate astrogliosis and inflammation following SCI.^19,37^ Similarly, we found that the GFAP+ area was significantly lower after oNPC transplantation in this study, thus, suggesting that the oNPCs contributed to a reduction of the glial scar.

The use of hiPSCs as the cell source for the present study introduces a potential risk for tumorigenesis.^31,38^ Notably, hiPSC-derived cells undergo numerous mitotic cycles prior to their application in a clinical setting. This may lead to the accumulation of genetic aberrations, such as genomic copy number variants,^39^ and it may furthermore impact the therapeutic efficacy of the transplanted cells. Therefore, it is imperative to test for tumorigenicity to ensure the safe clinical application of hiPSC-derived cells. In this study, we discovered that only 2.67 ± 0.48% of the GFP+ transplanted cells stained positive for Ki67+. Ki67 is a commonly used marker of dividing cells that is useful in diagnosing tumors.^40^ Notably, a Ki67 rate below 15% is classified as a low proliferative risk.^41^ Moreover, we did not observe any tumor formation at 8 months following transplantation, thus confirming the safety profile of the oNPCs.

When testing the cells in vivo, we aimed to use an animal model of SCI that would be suitable for clinical translation. The cervical clip-compression injury was chosen based on the prevalence of this type of injury in the SCI patient population. Although this particular model has been used in both mice and rats in our laboratory, rats display more neuropathological similarities to humans according to a preclinical-grading system,^42^ including pathophysiological features such as cavity formation, angiogenesis, and immunoreactions.^43,44^ Moreover, we used athymic RNU rats which lack T cells^45,46^ in order to circumvent immunorejection of the human cells.

The current study presents promising findings regarding the potential application of oNPCs for cervical SCI. However, future studies about whether oNPC-mediated remyelination contributes to electrophysiological improvements are warranted. In addition, future analyses should assess whether these transplanted cells are capable of promoting circuit formation with functional synaptic transmission. Finally, assessments of the various neurotrophic factors that may be expressed by oNPCs will provide additional valuable information regarding the role that the cells play in SCI.

## Conclusion

In the current study, we presented novel findings suggesting that hiPSC-oNPCs are a promising cell source for cervical SCI treatment. We observed that these cells predominantly differentiated into oligodendrocytes while retaining tripotency. Furthermore, the iPSC-oNPCs contributed to remyelination and were associated with reduced cavity volumes, decreased astrogliosis, enhanced axonal preservation, and improved functional recovery compared to vehicle treatment. Consequently, oNPCs demonstrate the potential to target multiple pathophysiological aspects of cervical SCI.

## Supplementary Material

szad044_suppl_Supplementary_Material

## Data Availability

Data are available from the corresponding authors on request.

## References

[1] Alizadeh A , Dyck SM, Karimi-Abdolrezaee S. Myelin damage and repair in pathologic CNS: challenges and prospects. Front Mol Neurosci. 2015;8(35). 10.3389/fnmol.2015.00035 PMC4515562 26283909

[2] Kanno H , Ozawa H, Tateda S, Yahata K, Itoi E. Upregulation of the receptor-interacting protein 3 expression and involvement in neural tissue damage after spinal cord injury in mice. BMC Neurosci. 2015;16:62. 10.1186/s12868-015-0204-0 26450067 PMC4599321

[3] Papastefanaki F , Matsas R. From demyelination to remyelination: the road toward therapies for spinal cord injury. Glia. 2015;63(7):1101-1125. 10.1002/glia.22809 25731941

[4] Akay LA , Effenberger AH, Tsai L-H. Cell of all trades: oligodendrocyte precursor cells in synaptic, vascular, and immune function. Genes Dev. 2021;35(3-4):180-198. 10.1101/gad.344218.120 33526585 PMC7849363

[5] Bankston AN , Mandler MD, Feng Y. Oligodendroglia and neurotrophic factors in neurodegeneration. Neurosci Bull. 2013;29(2):216-228. 10.1007/s12264-013-1321-3 23558590 PMC4020141

[6] Fernandez-Castaneda A , Gaultier A. Adult oligodendrocyte progenitor cells - multifaceted regulators of the CNS in health and disease. Brain Behav Immun. 2016;57:1-7. 10.1016/j.bbi.2016.01.005 26796621 PMC4940337

[7] Suárez-Pozos E , Thomason EJ, Fuss B. Glutamate transporters: expression and function in oligodendrocytes. Neurochem Res. 2020;45(3):551-560. 10.1007/s11064-018-02708-x 30628017 PMC6616022

[8] Hill CE , Proschel C, Noble M, et al. Acute transplantation of glial-restricted precursor cells into spinal cord contusion injuries: survival, differentiation, and effects on lesion environment and axonal regeneration. Exp Neurol. 2004;190(2):289-310. 10.1016/j.expneurol.2004.05.043 15530870

[9] Khazaei M , Ahuja CS, Fehlings MG. Generation of oligodendrogenic spinal neural progenitor cells from human induced pluripotent stem cells. Curr Protoc Stem Cell Biol. 2017;42:2D.20.1-2D.20.14. 10.1002/cpsc.31 28806852

[10] Nagoshi N , Khazaei M, Ahlfors J-E, et al. Human spinal oligodendrogenic neural progenitor cells promote functional recovery after spinal cord injury by axonal remyelination and tissue sparing: oligodendrogenic NPCs for spinal cord injury. Stem Cells Transl Med. 2018;7(11):806-818. 10.1002/sctm.17-0269 30085415 PMC6216444

[11] Nori S , Khazaei M, Ahuja CS, et al. Human oligodendrogenic neural progenitor cells delivered with chondroitinase ABC facilitate functional repair of chronic spinal cord injury. Stem Cell Rep. 2018;11(6):1433-1448. 10.1016/j.stemcr.2018.10.017 PMC6294173 30472009

[12] Ahuja CS , Wilson JR, Nori S, et al. Traumatic spinal cord injury. Nat Rev Dis Primers. 2017;3:17018. 10.1038/nrdp.2017.18 28447605

[13] Duval T , Saliani A, Nami H, et al. Axons morphometry in the human spinal cord. Neuroimage. 2019;185:119-128. 10.1016/j.neuroimage.2018.10.033 30326296

[14] Henmar S , Simonsen EB, Berg RW. What are the gray and white matter volumes of the human spinal cord?. J Neurophysiol. 2020;124(6):1792-1797. 10.1152/jn.00413.2020 33085549

[15] Saliani A , Zaimi A, Nami H, et al. Construction of a rat spinal cord atlas of axon morphometry. Neuroimage. 2019;202:116156. 10.1016/j.neuroimage.2019.116156 31491525

[16] Forgione N , Karadimas SK, Foltz WD, et al. Bilateral contusion-compression model of incomplete traumatic cervical spinal cord injury. J Neurotrauma. 2014;31(21):1776-1788. 10.1089/neu.2014.3388 24949719 PMC4186801

[17] Chambers SM , Fasano CA, Papapetrou EP, et al. Highly efficient neural conversion of human ES and iPS cells by dual inhibition of SMAD signaling. Nat Biotechnol. 2009;27(3):275-280. 10.1038/nbt.1529 19252484 PMC2756723

[18] Shibata S , Murota Y, Nishimoto Y, et al. Immuno-electron microscopy and electron microscopic in situ hybridization for visualizing piRNA biogenesis bodies in drosophila ovaries. Methods Mol Biol. 2015;1328:163-178. 10.1007/978-1-4939-2851-4_12 26324437

[19] Wilcox JT , Satkunendrarajah K, Zuccato JA, Nassiri F, Fehlings MG. Neural precursor cell transplantation enhances functional recovery and reduces astrogliosis in bilateral compressive/contusive cervical spinal cord injury: neural stem cells improve recovery in cervical SCI. Stem Cells Transl Med. 2014;3(10):1148-1159. 10.5966/sctm.2014-0029 25107585 PMC4181397

[20] Soubeyrand M , Badner A, Vawda R, Chung YS, Fehlings MG. Very high resolution ultrasound imaging for real-time quantitative visualization of vascular disruption after spinal cord injury. J Neurotrauma. 2014;31(21):1767-1775. 10.1089/neu.2013.3319 24831774 PMC4186763

[21] Moonen G , Satkunendrarajah K, Wilcox JT, et al. A new acute impact-compression lumbar spinal cord injury model in the rodent. J Neurotrauma. 2016;33(3):278-289. 10.1089/neu.2015.3937 26414192 PMC4744888

[22] Iwasaki M , Wilcox JT, Nishimura Y, et al. Synergistic effects of self-assembling peptide and neural stem/progenitor cells to promote tissue repair and forelimb functional recovery in cervical spinal cord injury. Biomaterials. 2014;35(9):2617-2629. 10.1016/j.biomaterials.2013.12.019 24406216

[23] Hofstetter CP , Holmström NAV, Lilja JA, et al. Allodynia limits the usefulness of intraspinal neural stem cell grafts; directed differentiation improves outcome. Nat Neurosci. 2005;8(3):346-353. 10.1038/nn1405 15711542

[24] Yousefifard M , Rahimi-Movaghar V, Nasirinezhad F, et al. Neural stem/progenitor cell transplantation for spinal cord injury treatment; a systematic review and meta-analysis. Neuroscience. 2016;322:377-397. 10.1016/j.neuroscience.2016.02.034 26917272

[25] Sharif-Alhoseini M , Khormali M, Rezaei M, et al. Animal models of spinal cord injury: a systematic review. Spinal Cord. 2017;55(8):714-721. 10.1038/sc.2016.187 28117332

[26] Karimi-Abdolrezaee S , Eftekharpour E, Wang J, Morshead CM, Fehlings MG. Delayed transplantation of adult neural precursor cells promotes remyelination and functional neurological recovery after spinal cord injury. J Neurosci. 2006;26(13):3377-3389. 10.1523/JNEUROSCI.4184-05.2006 16571744 PMC6673854

[27] Nashmi R , Fehlings MG. Changes in axonal physiology and morphology after chronic compressive injury of the rat thoracic spinal cord. Neuroscience. 2001;104(1):235-251. 10.1016/s0306-4522(01)00009-4 11311546

[28] Karimi-Abdolrezaee S , Eftekharpour E, Fehlings MG. Temporal and spatial patterns of Kv1.1 and Kv1.2 protein and gene expression in spinal cord white matter after acute and chronic spinal cord injury in rats: implications for axonal pathophysiology after neurotrauma. Eur J Neurosci. 2004;19(3):577-589. 10.1111/j.0953-816x.2004.03164.x 14984408

[29] Fehlings MG , Nashmi R. Assessment of axonal dysfunction in an in vitro model of acute compressive injury to adult rat spinal cord axons. Brain Res. 1995;677(2):291-299. 10.1016/0006-8993(95)00141-c 7552255

[30] Cummings BJ , Uchida N, Tamaki SJ, et al. Human neural stem cells differentiate and promote locomotor recovery in spinal cord-injured mice. Proc Natl Acad Sci USA. 2005;102(39):14069-14074. 10.1073/pnas.0507063102 16172374 PMC1216836

[31] Nori S , Okada Y, Yasuda A, et al. Grafted human-induced pluripotent stem-cell-derived neurospheres promote motor functional recovery after spinal cord injury in mice. Proc Natl Acad Sci USA. 2011;108(40):16825-16830. 10.1073/pnas.1108077108 21949375 PMC3189018

[32] Abematsu M , Tsujimura K, Yamano M, et al. Neurons derived from transplanted neural stem cells restore disrupted neuronal circuitry in a mouse model of spinal cord injury. J Clin Invest. 2010;120(9):3255-3266. 10.1172/JCI42957 20714104 PMC2929730

[33] Aimone JB , Leasure JL, Perreau VM, Thallmair M. Spatial and temporal gene expression profiling of the contused rat spinal cord. Exp Neurol. 2004;189(2):204-221. 10.1016/j.expneurol.2004.05.042 15380473

[34] Gong L , Gu Y, Han X, et al. Spatiotemporal dynamics of the molecular expression pattern and intercellular interactions in the glial scar response to spinal cord injury. Neurosci Bull. 2023;39(2):213-244. 10.1007/s12264-022-00897-8 35788904 PMC9905408

[35] Lau LW , Keough MB, Haylock-Jacobs S, et al. Chondroitin sulfate proteoglycans in demyelinated lesions impair remyelination. Ann Neurol. 2012;72(3):419-432. 10.1002/ana.23599 23034914

[36] Wei Y , Andrews MR. Advances in chondroitinase delivery for spinal cord repair. J Integr Neurosci. 2022;21(4):118. 10.31083/j.jin2104118 35864769

[37] Kumagai G , Tsoulfas P, Toh S, et al. Genetically modified mesenchymal stem cells (MSCs) promote axonal regeneration and prevent hypersensitivity after spinal cord injury. Exp Neurol. 2013;248:369-380. 10.1016/j.expneurol.2013.06.028 23856436

[38] Nori S , Okada Y, Nishimura S, et al. Long-term safety issues of iPSC-based cell therapy in a spinal cord injury model: oncogenic transformation with epithelial-mesenchymal transition. Stem Cell Rep. 2015;4(3):360-373. 10.1016/j.stemcr.2015.01.006 PMC4375796 25684226

[39] Yamamoto T , Sato Y, Yasuda S, et al. Correlation between genetic abnormalities in induced pluripotent stem cell-derivatives and abnormal tissue formation in tumorigenicity tests. Stem Cells Transl Med. 2022;11(5):527-538. 10.1093/stcltm/szac014 35445254 PMC9154342

[40] Li LT , Jiang G, Chen Q, Zheng JN. Ki67 is a promising molecular target in the diagnosis of cancer (review). Mol Med Rep. 2015;11(3):1566-1572. 10.3892/mmr.2014.2914 25384676

[41] Jonat W , Arnold N. Is the Ki-67 labelling index ready for clinical use?. Ann Oncol. 2011;22(3):500-502. 10.1093/annonc/mdq732 21343384

[42] Kwon BK , Okon EB, Tsai E, et al. A grading system to evaluate objectively the strength of pre-clinical data of acute neuroprotective therapies for clinical translation in spinal cord injury. J Neurotrauma. 2011;28(8):1525-1543. 10.1089/neu.2010.1296 20507235 PMC3143387

[43] Byrnes KR , Fricke ST, Faden AI. Neuropathological differences between rats and mice after spinal cord injury. J Magn Reson Imaging. 2010;32(4):836-846. 10.1002/jmri.22323 20882614 PMC2949295

[44] Surey S , Berry M, Logan A, Bicknell R, Ahmed Z. Differential cavitation, angiogenesis and wound-healing responses in injured mouse and rat spinal cords. Neuroscience. 2014;275:62-80. 10.1016/j.neuroscience.2014.06.003 24929066

[45] Piltti KM , Salazar DL, Uchida N, Cummings BJ, Anderson AJ. Safety of epicenter versus intact parenchyma as a transplantation site for human neural stem cells for spinal cord injury therapy. Stem Cells Transl Med. 2013;2(3):204-216. 10.5966/sctm.2012-0110 23413374 PMC3659765

[46] Piltti KM , Salazar DL, Uchida N, Cummings BJ, Anderson AJ. Safety of human neural stem cell transplantation in chronic spinal cord injury. Stem Cells Transl Med. 2013;2(12):961-974. 10.5966/sctm.2013-0064 24191264 PMC3841091

