# Supplementary material for: Human Spinal Oligodendrogenic Neural Progenitor Cells Enhance Pathophysiological Outcomes and Functional Recovery in a Clinically Relevant Cervical Spinal Cord Injury Rat Model
Source: Stem Cells Transl Med. 2023 Aug 24;12(9):603–16. doi: 10.1093/stcltm/szad044 (PMC10502566; doi:10.1093/stcltm/szad044)
Supplement: szad044_suppl_Supplementary_Material [file szad044_suppl_supplementary_material.pdf]

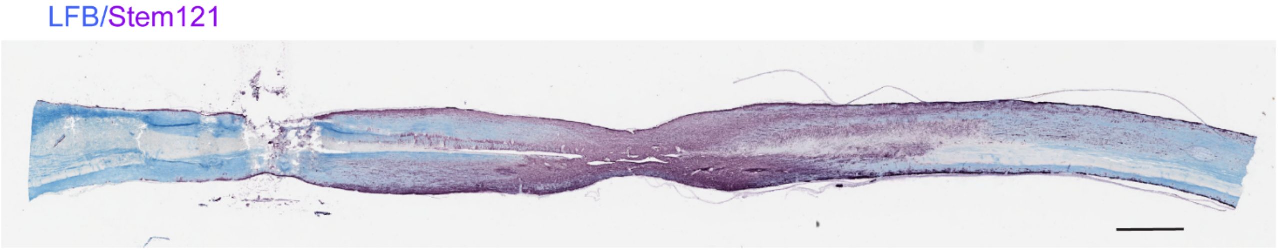

Supplemental Figure 1.

Two weeks following cervical SCI, oNPCs were transplanted into RNU rats. After eight months, the animals were sacrificed, and tissue samples were collected for analysis. Immunohistochemistry was performed using the Stem121 antibody, which specifically labels human cells within rat tissue. Subsequently, the samples were treated with an HRP-conjugated secondary antibody and developed using NovaRed (Vector Laboratories). Luxol Fast Blue (LFB) was used for counterstaining of the tissues. Notably, no signs of tumorigenicity were observed in the spinal cords of any animals. Scale bar = 1500 μm.

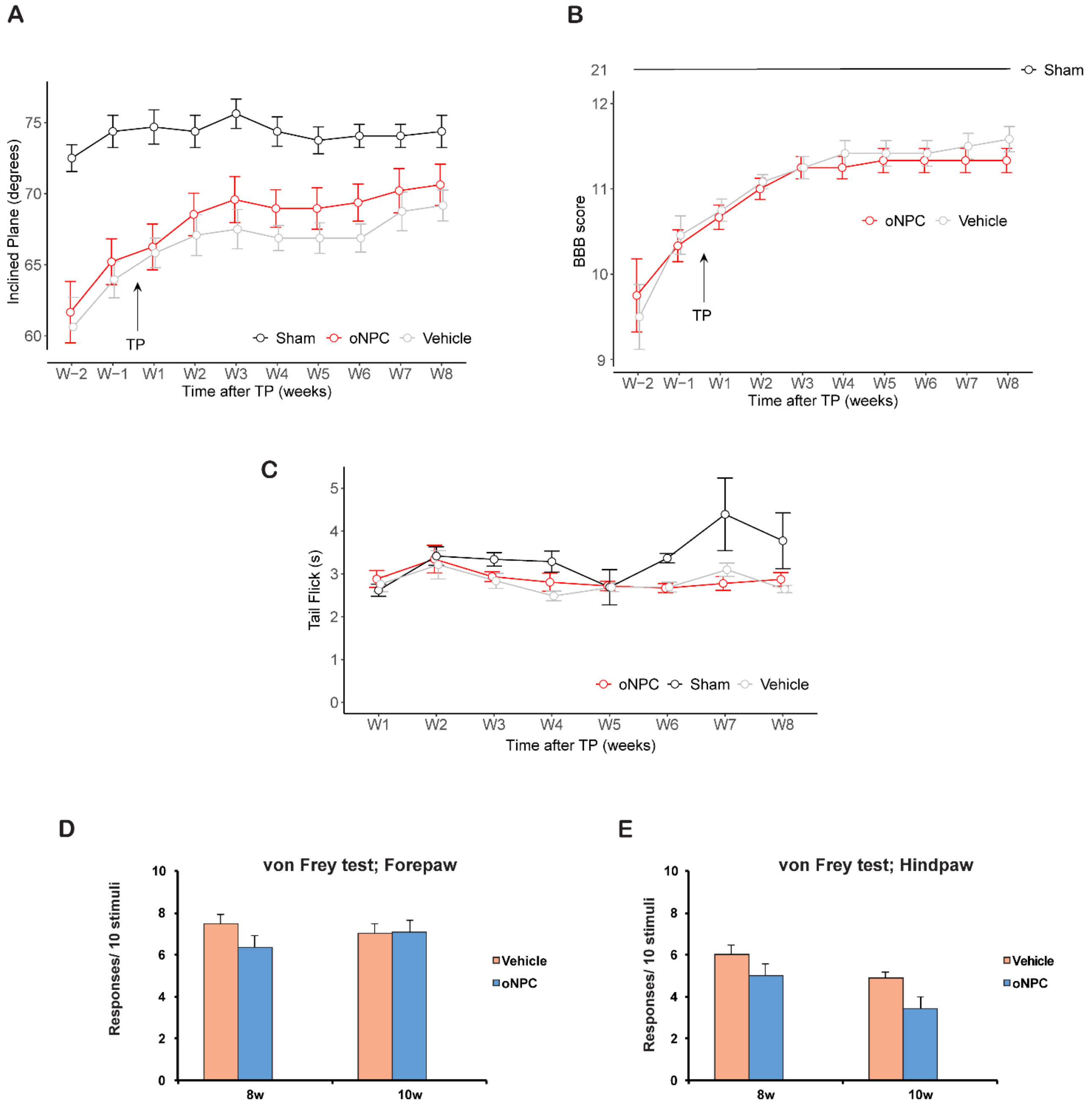

Supplemental Figure 2.

Additional functional and pain analyses following oNPC transplantation. (A and B) There were no significant differences in the inclined plane test or BBB score between the groups. (C) There was no significant difference detected between the groups when when evaluating for thermal allodynia using the tail-flick test. (D and E) Evaluation of mechanical allodynia using the von Frey test in the forepaw (D) and hindpaw (E) at 8 and 10 weeks following SCI. The von Frey test responses were not significantly different between the groups.
